# Early evaluation of experiences of health care providers in reception centers with a patient-held personal health record for asylum seekers: a multi-sited qualitative study in a German federal state

**DOI:** 10.1186/s12992-018-0394-1

**Published:** 2018-07-20

**Authors:** Rosa Jahn, Sandra Ziegler, Stefan Nöst, Sandra Claudia Gewalt, Cornelia Straßner, Kayvan Bozorgmehr

**Affiliations:** Department of General Practice and Health Services Research, University Hospital Heidelberg, Im Neuenheimer Feld 130.3, 69120 Heidelberg, Germany

**Keywords:** Human migration, Continuity of patient care, Asylum seekers, Refugees, Quality of health care, Qualitative research

## Abstract

**Background:**

The provision of high-quality medical care to asylum seekers represents a key challenge in many countries of the European Union. Especially continuity of care has been difficult to achieve as the migrant trajectory moves asylum seekers across and within European countries. Patient-held personal health records (PHR) have been proposed to facilitate the transfer of medical history between health sectors and providers, but so far there is no data to support its use in the migrant setting. The present paper addresses this knowledge gap by exploring the experiences and practices of healthcare providers in reception centers for asylum seekers using a patient-held PHR as well as the perceived associated benefits and shortcomings.

**Methods:**

Early evaluation by means of a multi-sited qualitative study in six asylum seeker reception centers in five cities in the German state of Baden-Wuerttemberg, conducted between November 2016 and January 2017. The PHR evaluated in this study was implemented in five of these reception centers between February and October 2016; the remaining one only receiving patients with the PHR through transfer from the other facilities. 17 interviews were conducted with physicians and nurses working at these reception centers exploring their experiences, routines, and perspectives regarding the patient-held PHR. The interviews were recorded, transcribed and analyzed following the approach of thematic analysis.

**Results:**

Healthcare providers recognise the potential of a patient-held PHR to improve access to medical history. They use the PHR to document their medical consultations and to collect other medical reports. However, physician adherence to the patient-held PHR was described as unsatisfactory, in particular among external doctors, thus limiting its immediate benefit. Reasons given for this low adherence included lack of information before implementation, demanding working conditions with little support, low perceived benefits depending on the degree of fragmentation of settings, parallel existence of other documentation platforms and strained patient relationships.

**Conclusion:**

A patient-held PHR could improve the availability of health-related information in reception centers if a context-sensitive implementation process achieves high adherence to the PHR among physicians as well as high patient compliance and includes guidelines regarding its adequate integration into local routines.

**Electronic supplementary material:**

The online version of this article (10.1186/s12992-018-0394-1) contains supplementary material, which is available to authorized users.

## Background

In autumn 2015, the European Union recorded up to 180,000 asylum applications per month [1]. Although numbers have dropped to roughly a third since then, providing asylum seekers with access to appropriate high-quality medical care continues to be problematic. One of the key challenges has been the continuity of medical care, namely establishing communication and networks between health care providers treating refugees and asylum seekers as their trajectory continues to move them within and across different European countries [2, 3].

In Germany, initial provision and organization of medical care to asylum seekers falls under the responsibilities of state-level reception centers. These centers accommodate asylum seekers upon their entry into Germany for six months before they are transferred to peripheral reception centers in other federal states, or districts and communities. Depending on their country of origin, asylum seekers can be retained in state-level reception centers considerably longer or until the end of the asylum procedure. Furthermore, asylum seekers are screened for infectious diseases in state-level reception centers before any transfer is initiated [4]. The organization of care delivery in reception centers is not regulated by national or state-level guidelines and follows different procedures at every center [5]. A recent national survey of public health authorities has found that the lack of intersectoral communication is a major obstacle to the delivery of high-quality care to asylum seekers in this fragmented and heterogeneous setting. The study concluded that, in the absence of national guidelines and unified electronic health records, a standardized, patient-held Personal Health Record (PHR) represents the only viable means to transmit health-related information between providers and across sectors [6].

However, patient-held PHRs have only been evaluated in routine care settings in areas such as obstetrics, oncology, psychology, palliative care and chronic diseases [7–16]. So far, no study has evaluated PHRs in the provision of medical care to refugees or asylum seekers. Thus, it remains unclear whether a patient-held PHR improves the transfer of health-related information during the migrant trajectory, how such a medium is used by health care providers and asylum seekers, and what barriers and enablers affect the successful implementation of patient-held PHRs in reception centers.

This paper presents (i) the experiences of health care providers in reception centers using a patient-held PHR in a German federal state, (ii) their local practices in using the PHR within the routine of care provision and (iii) their perspective on perceived benefits and shortcomings of the patient-held PHR with respect to both availability and transfer of health-related information.

## Methods

Between November 2016 and January 2017, we conducted a multi-sited qualitative study of healthcare providers’ experiences with a recently introduced patient-held PHR in reception centers in Baden-Wuerttemberg, Southern Germany.

### Development and content of the PHR

In October 2015, the Department of General Practice and Health Services Research at the University Hospital Heidelberg, in cooperation with health care providers in the state’s main reception and registration center (Patrick-Henry-Village, PHV) initiated the bottom-up development of a patient-held PHR [17]. The process was based on pre-existing documentation forms at the University Hospital Heidelberg as well as material developed by a publisher of medical pictograms (Setzer Verlag “Tip Doc” [18]) and a local network on medication safety (Aktionsbündnis Rhein-Neckar, “Mein Plan” [19]). To ensure the PHR would be viable in the clinical setting of reception centers, clinicians were consulted to provide input on the first draft and develop additional, generic documentation forms to be included in the final version [17]. The content and format of the PHR were subsequently discussed and agreed within the steering committee of the reception center. The final version of the PHR (version 1.0) was a small booklet (148 × 210 mm) with 15 pages comprising (i) patient information about the PHR in ten languages (Albanian, Arabic, Dari/Farsi, German, French, Romanian, Russian, Serbian, Tigrinya, and Urdu), (ii) explanations for doctors on how to use the PHR, (iii) a document pocket to insert documents such as vaccination cards, (iv) a table for chronic diseases and pre-existing conditions, (v) a medication plan for long-term medication, (vi) a table for the continuous documentation of consultations, (vii) a table for test results and (viii) a multilingual table for upcoming medical appointments. The PHR was meant for universal use by all patients, and its contents did not differ for patients of different age, gender or by medical conditions.

### Implementation of the PHR

The PHR was initially created to facilitate communication between providers in PHV, external hospitals, and private practices. The first version of the patient-held PHR was introduced as a pilot in February 2016 in PHV and has been positively received by medical staff working at the reception center. Interim feedbacks were reported to the team involved in designing the card and minor changes in format and content were performed to increase practicability in every-day handling. Encouraged by the positive feedback four months after the initial piloting and routine use in PHV, the state of Baden-Wuerttemberg subsequently provided funding for the PHR and expanded it to five of the twelve other reception centers in the state. The state authorities published an announcement in national and local medical newspapers and the Department of General Practice and Health Services Research sent an information letter to all doctors in the surrounding area as to inform all medical staff who might come into contact with the PHR about its use and purpose. Local state authorities and health care providers in reception centers were responsible for both implementation and continuous use of the PHR.

### Setting and study context

Before PHR implementation, each reception center had developed its routine of care delivery and documentation [5]. While some reception centers were using one or more electronic health record systems, others worked with paper-based case files or none at all. The reception centers provided medical care through on-site services and only transferred patients to specialized practitioners in private practices or hospitals (hereafter “external doctors”) if medically indicated. The number of medical staff in reception centers varied from less than ten to several dozen doctors involved in the provision of care. Some reception centers only provided primary care by general practitioners, while others additionally offered a range of services including pediatrics, obstetrics, midwifery care, and psychosocial services. The doctors were either employed by cooperating hospitals or commercial or not-for-profit medical contractors. In most study sites, physicians were supported by nurses who were involved in providing medical care and often tasked with the medical departments’ administration. The number of patients treated per day ranged from less than 10 (at the smallest reception center) to 80 and more per day (at the largest reception center) during the study period (personal communication with state authorities).

### Study population and study sites

We conducted 17 semi-structured interviews with 11 doctors (interview codes: D1–11) and six nurses (N1–6) in six reception centers across four cities in Baden-Wuerttemberg one to three months after the introduction of the PHR for an early evaluation. The research team recruited interview participants at five reception centers selected for PHR implementation by state level authorities. In addition, two interviews were conducted with medical staff at a sixth reception center which had not introduced the PHR yet but had been receiving asylum seekers with PHRs from the five other reception centers.

### Participant selection and data collection

A purposive sampling strategy was adopted, aiming for diversity among participants regarding medical specialty and work experience in the reception center setting. We approached participants personally, informing them about purpose and content of the study. After written consent was obtained, we scheduled interviews with participants at their work places. The interviews explored the perceptions regarding the availability of health-related information before and after PHR introduction, its utilization by the participants and their colleagues, PHR integration into the work flows of care provision as well as its benefits and limitations. The interview guide was developed in repeated discussions within the research team consisting of all authors of this manuscript and was slightly adapted after a first pilot interview (see Additional file 1 for final interview guide). The semi-structured interview was followed by a moderated think aloud section, in which the participant read and commented on the PHR page by page. Interviews were conducted by SZ, an interdisciplinary anthropologist, and RJ, a researcher and physician. All interviews were carried out in German and lasted 15 min to one hour. Interviews were recorded digitally and transcribed verbatim using the F4 transcription software. Additionally, the interviewers recorded qualitative memos and observations in field notes.

### Data analysis

A thematic analysis was performed, based on the methodology described by Holliday (2007) [20]. Interview transcripts, field notes and think aloud protocols were discussed within the research team. Emergent themes were captured in an initial coding system. Transcripts were subsequently coded in MAXQDA by RJ, gradually modifying the code system to fully capture all themes emerging from the data (for code system, see Additional file 2). The resulting codes and codings were then discussed by the interviewers and the research team to develop an intersubjective understanding of experiences and realities revealed by the interviews. Quotes presented in the results section of this paper were translated by a bilingual researcher at the department.

## Results

### Participant characteristics

The study sample included eleven physicians, 38 to 75 years of age, ten working in primary care and one in psychosocial care in the reception centers. They reported 30 to several hundred patient contacts in this setting per week and have been providing care for asylum seekers seven months to seventeen years. Most physicians were additionally working in their own practices or at university hospitals in the region. The six nurses included in the study (35 to 58 years of age) were employed either part time or full time at the reception centers. Most had worked in hospitals or private practices prior to working in the reception center.

### Availability of health-related information

The participating physicians unanimously regarded knowledge about a patient’s medical history as crucial to the delivery of high-quality care and emphasized its special relevance in the reception center setting. They explained that their consultations and therapies were particularly reliant on the availability of medical health records because obtaining information from the patient is often impeded by language barriers. However, they reported problems in access to medical history stemming from consultations both inside and outside the reception center.

“[…] that’s the crux of the work out there, that it is so difficult to get information, above all because of the interpreters and language situation” (D11).

Access to written information about consultations by colleagues within the reception center varied between study sites, largely because each had adopted a different electronic or paper-based health record system. Participants from the largest reception center (PHV) described that two different electronic health record systems had been implemented, each used by a different group of doctors and without access to or integration with the other. This was reportedly due to sectoral and legal barriers regarding physicians’ organizational affiliation. Participants working at PHV bemoaned the effect of this situation on access to medical history:

“Sometimes we are really at a loss because the health record systems are so different [from one another] [...] with regards to access to information this is a catastrophe” (D11).

Participants from reception centers with a small number of medical staff appeared to be more satisfied with the current state of internal communication.

Information about external consultations was reported to be often unavailable. Study participants said that patients rarely bring back written documentation after external consultations and external doctors often did not send reports to the reception centers’ medical departments. This would result in lengthy searches to find out where the patient had been and phone calls with the respective doctor to ask for medical information.

“What we often see is that, he [the patient] has seen a dermatologist, but which one. A urologist, in the hospital, [or] in a private practice, where did he end up? Nobody knows, until we’ve called around, or found the right person at the coordination desk, someone, who has the time, to ask around, make phone calls and ask where he [the patient] has been” (D1).

To remedy this problem, participants from several reception centers had painstakingly developed networks of cooperative doctors.

“Yes, we send patients preferably to doctors that provide us with documents. We have a list of doctors […] to whom we send [patients] […] they send us [their] doctor’s notes” (D5).

“It was hard work to establish such a network [of cooperative doctors], and it was critical [for the availability of medical reports from external doctors]” (D6).

No interview reported patients bringing personal health records from their country of origin. Some, however, described rare instances of doctor’s notes from the patient’s country of origin or from transit countries such as Turkey.

“Then they show something in Turkish or Kurdish and then, well… I can’t really do much with that. Except for the medical terms, yes, if it says “haemoglobin”, then I can recognize that” (D9).

### PHR implementation – Process and barriers

The health care personnel described the first weeks after PHR introduction as a transition and adaptation period, in which they needed to get used to the new tool and overcome emerging barriers to its integration into their daily routine (for a summary of implementation challenges and facilitators, see Table 1).

**Table 1:** Implementation challenges and facilitators

Major Theme	Implementation Challenges	Implementation Facilitators
Working conditions	High demand, low support	Supportive working environment
	Stress, high work load	Support by nurses
	Limited resources	Support by interns/students
	Strained relationships with patients	
Patient management	Low patient adherence to the PHR	High patient adherence to the PHR
	New PHRs are handed out to the same patient multiple times	Encouraging patients to retrieve their PHR in case they have forgotten
	Patients do not receive appropriate information about the PHR	Patients receive appropriate information about the PHR and understand the relevance for their medical treatment
Local PHR practices	Low physician adherence to the PHR	High physician adherence to the PHR
	Physicians receive no or insufficient information about the PHR before implementation	Strong involvement by nurses, e.g. preparing the PHR prior to the consultation
	Documenting in multiple paper-based or electronic health records	Printing electronic PHR and storing it in the patient-held PHR’s document pocket to lower workload
	Illegible handwriting	Using the PHR as a folder for all relevant documents
Potential benefit of a patient-held PHR	Low perceived benefit in settings of low fragmentation	High perceived benefit in settings of high fragmentation
	Well-established electronic PHR accessible to all health care providers in the facility	Absence of electronic PHR or more than one electronic PHR system
	Small number of personnel	Large number of personnel
	Close collaboration and personal communication with external doctors prior to PHR introduction	Dissatisfaction with availability of medical history and communication with external doctors prior to PHR introduction
	Mono-disciplinary care settings	Different professions and medical specialties involved in care provision

PHR: patient-held personal health record

Many study participants expressed that they had not received sufficient information about the PHR before its introduction, adding to the difficulties of those first weeks. In fact, one physician who only works at the reception center on weekends did not know what the PHR was until he was recruited to participate in the interviews for its evaluation.

“The doctors had to get accustomed to writing in them [the PHRs], […] there was a bit of an adjustment period” (N3).

“Many of us [doctors] didn’t know that this personal health record exists at all or what it is for exactly and well maybe that’s also why it was difficult in the beginning” (D1).

It emerged from the interviews that working conditions in the reception centers were perceived to be a key barrier to the implementation of a patient-held PHR. Many participants, mainly physicians, described their work environment as stressful and intense. Some talked about tensions between staff and patients and a feeling of being left alone, without the necessary resources to do their job properly.

“Here I do not receive any support, […] and that’s why I don’t do it often because it can be simply fatal to have to do everything alone from 10 to 6 o’clock” (D9).

“We’re there for six, seven hours and get headaches figuratively speaking, headaches from [working with] the asylum seekers, headaches from the security personnel, headaches from the nurses that come in every time, and yes – headaches from the documentation” (D4).

In this context, the additional workload caused by the PHR was seen as a major obstacle to its implementation by many participating physicians. This was especially so where reception centers had a well-established electronic PHR in place. In these cases, introducing the patient-held PHR meant that the patient’s medical information had to be recorded on two systems, increasing the documentation workload and, in some cases, leading to confusion between the systems.

“I have to look at it again on [electronic health record], [...] because one colleague reports in here and the other documents in there and the third documents in both and the fourth does not have any time to document anything but there is a doctors’ note, that is often chaotic” (D6).

“You have to document everything, on the computer, for the institution itself, and now also for the PHR. It is a triple burden of paperwork.” (D4).

The burden of using the patient-held PHR appeared to be abetted in the presence of ancillary health workers, students or interns that were able to help out with the documentation.

“Whenever there are interns or students with us, or someone helps out, you can basically document simultaneously in the computer and the other [person] copies it straight from there into the booklet [...] that works, too” (D1).

### The PHR routine

In all but one study site, the PHR was handed out to patients by a nurse or a doctor. In some cases, nurses prepared the booklet with the patient’s name and ID to expedite the process. In one reception center, the PHR was handed out and the first entries were made by the public health service that conducts the mandatory medical entry screening and provides vaccinations.

Handing out the PHR did not necessarily include explaining the purpose of the PHR to patients. None of the study reception centers had established standardized procedures regarding the briefing of patients about the PHR. In one case, the nurses believed that the doctors would inform patients and the doctors thought the opposite was the case, leaving patients with little or no knowledge about the PHR. The form and content of the explanation provided varied but often included highlighting the importance of the PHR for the provision of safe and continuous care.

“You just have to explain it differently, so that they understand it and the booklet [exists] so that you don’t get the wrong medication and die” (N2).

“We [doctors] tell them [asylum seekers] that they always have to take it with them. Yes, that it’s simply really important for the documentation and success of the therapy” (D7).

At most of the study sites, healthcare staff had put in place mechanisms enforcing patient adherence to the PHR. These often included rules regarding the treatment of patients who did not take their PHR with them to medical appointments. Most participants, doctors, and nurses, sent patients back to their accommodation to fetch the PHR before offering care. One doctor described that she gave the security men at her door a sample PHR and told them to check if the patients have theirs with them.

“We really tell them [asylum seekers] to go get it [PHR]. Yes yes, that is a little bit of an educational exercise” (N3).

“I really find it most sensible that no one treats a patient who doesn’t bring the booklet along” (D6).

Another participant described a different routine he observed at his reception center, where patients simply got a new PHR whenever they forgot to bring theirs along – a practice that was regarded as undermining the aim of the PHR to facilitate information transfer.

“What do the nurses do, well they give him [asylum seeker] a new one [...] but the refugees are not stupid either, why should I take it with me if I get a new one anyway” (D4).

### Use and non-use of the personal health record

The physicians interviewed for this study agreed that they use the PHR to obtain information on a patient’s medical history. However, it was repeatedly pointed out that the relevance of the PHR within a consultation varied by patient and symptoms.

“[..] it depends, multimorbid, chronically sick [patients] [...] you would always look at it [PHR] [...] But very young patients, coming in with a common cold or back pain again and again, I don’t […] fully read everything that has been written in there.” (D1).

While expressing that the PHR was particularly important for complex chronic or oncologic conditions, some respondents noted that it was not suitable to carry enough information for the actual therapy and monitoring of these patients.

“Let’s assume that we have a patient with a long medical history, and next week he has to be treated by an external doctor. […]But then the information that is in the personal health record is too thin – to really [do] something well informed there” (D11).

Regarding the documentation of their own consultations, most participants said they record at least diagnoses and prescriptions as well as relevant test results such as blood pressure. Some physicians also recorded laboratory results, while others admitted forgetting to use the PHR altogether when under stress.

“In the health booklet I limit myself to diagnosis and therapy. Or maybe temperature, blood pressure et cetera whenever it would make sense [to include]” (D1).

“I do that [documentation] very briefly sometimes. A key word, “everything is okay”” (D2).

“[I use the PHR] if it is there and if I remember it I must confess” (D11).

The views regarding the use of the patient-held PHR by internal colleagues diverged substantively. Participants from two study sites said that they usually saw their colleagues’ notes in the PHR and believe that most of them did document their consultations regularly. At one study site, a participant was much more skeptical, describing that most of her colleagues did not use the patient-held PHR at all. In the remaining two reception centers under study, the uptake of the PHR among internal physicians was described as heterogeneous, with some documenting their consultations in the PHR on a regular basis or printing reports from the electronic health records and others being more inconsistent in its use. Also, some participants described not being able to read their colleagues’ handwritings in the PHR.

“The majority of my colleagues, I see that in the booklets […] they [doctors] all write in there” (D3).

“I check the health booklet to see what is written there, if there is something in there at all, often there is nothing.” (D11).

The existence of parallel health record systems appeared to interrupt the uptake of the PHR within the reception centers as physicians had to document their consultations and access information on all available systems. Some doctors solved this apparent conflict by documenting their consultations in the electronic personal health record only, printing the electronic record and storing it in the PHR’s document pocket.

“I’ve gotten into the habit of writing only the date in the health booklet by hand, today, the first of December, see computer print-out.[…] The previous print-out is shredded, and the current one is kept in there [document pocket in the PHR]. For me, that is the documentation in the health booklet” (D3).

Interviewed nurses mainly used the PHR for carrying out tasks based on the information contained in the PHR without consulting a doctor.

“Long term medication, blood pressure medication, diabetes medication, where you know he [patient] always needs these, and then we can [...] immediately order the medication, and he does not need to see the doctor” (N1).

“It can happen that people are scheduled for the next days or [...] once a week, and then we look in here [PHR] [...] and know that he will have his blood taken and we can do that without the patient having to see the doctor, and he only returns with his results” (N1).

In one reception center, nurses also used the PHR to communicate what they perceived as particularly relevant information about the patients through color codes. They used colored stickers on the cover of the PHR to indicate that a patient has an infectious disease, a mental health issue or is pregnant. If the patient received anticoagulation such as Marcumar®, the PHR in this facility was marked with an “M”. A nurse explained this procedure as follows:

“It always makes sense [...] to know beforehand what we are dealing with [...] Sometimes not all [psychiatric patients] take their medication, and we had a few patients who freaked out regularly [...] now there is always someone at the door who sees this booklet and says aha, mentally ill, I will call my colleague [security staff] for general safety” (N5).

The PHR’s use appeared to be a lot more irregular among external doctors. Where individual interviewees remembered having read PHR entries by external doctors, these were noted as singular incidents and often by doctors with whom a close collaboration had already been established.

“No hospital writes in here [PHR]. They have discharge letters. [...] and no external doctor writes anything in here” (D5).

“I have rarely seen that -maybe two or three times that an external doctor [...] wrote anything in the health booklet” (D1).

In many cases, the reception centers still received written reports from external doctors which were then stored in the patient-held PHR’s document pocket. In this sense, the PHR was used as a storage place for all relevant personal medical documents. Highlighting the relevance of this function, many participants suggested that in the future, the PHR should include an additional document pocket or the format be changed from a booklet to an actual folder.

“They [the patients] take from one of their pockets some medical report that they received [from a doctor] somewhere, and then I tell them that needs to go in here [PHR], the booklet is basically a file folder” (D3).

While a few interviewees reported negative experiences with the patients’ treatment of the PHR, most believed that the majority of asylum seekers had a positive attitude towards it.

“It is not that we get really horrible booklets, well sometimes it is worn out, but overall it is being treated fairly well” (N3).

“Some [...] treat it like a really important document, an ID so to speak, and [...] others, with them it looks awful, food stains everywhere, coffee stains, I don’t know what – so there are both extremes” (D1).

### Benefits and limitations of the patient-held PHR

The perceived inconsistent utilization of the PHR, both by internal and external colleagues was reflected in the way participants assessed its benefits. During the interviews, participants discussed the benefits of the PHR both regarding actual, immediate changes in their work reality as well as hypothesized or potential advantages of the PHR for themselves, their colleagues and the patients once it would be implemented in daily routine. No participant doubted the general benefit of a PHR for the quality of care along the patients’ in-country journey.

“If this is really the plan […] that he [the patient] has a complete booklet, then for him simply the quality of care is improved” (N6).

“There is a point in the booklet if it is properly kept” (D4).

### Impact on internal transfer of information: Fragmentation and interprofessional care

The extent to which the study participants perceived a concrete benefit of the PHR for the transfer of information between doctors within one reception center was significantly influenced by the availability and design of electronic health records prior to PHR implementation. In reception centers that did not have one electronic health record system accessible to all doctors working in its medical departments, the PHR was perceived as an essential tool for the internal transfer of medical history. In these settings, too, the PHR’s benefit was hampered by inconsistent utilization.

“No one can look into the other [electronic health record]. For this, the booklet is the most important contact point of all” (N1).

“We ensure with it [the PHR] the continuity of care for other colleagues and for ourselves, well, without that it does not work at all” (D2).

“Most of the time I read only my own handwriting […] that is why the help I get from this booklet is zero” (D5).

Participants from the reception centers that used one electronic health record system believed the patient-held PHR to be less beneficial to their internal communication.

“I am telling you honestly primarily important for the doctors here is the electronic health record” (D4).

“Before we had the computer, the booklet, for me personally it was more important than now […] the computer […] gives me all the data that I need if he [the patient] has been here before” (D8).

Another benefit that emerged from the interviews was that the patient-held PHR improves interprofessional communication between physicians and nurses. As nurses could utilize information in the PHR, they needed less time consulting the doctor. Instead, they could answer minor questions patients might have or hand out prescriptions based on the information contained in the PHR.

“This is the big benefit of this booklet for us, if everything is really in there, we can get the prescriptions ready, and the people don’t have to see the doctor twice” (N1).

### Impact on information transfer across organizations: The case of external consultations

Prior to PHR implementation, access to information about external consultations had been a significant problem. The benefit of a PHR in bringing documentation of external consultations back to the reception center was generally acknowledged. However, the aforementioned low uptake among external doctors means that it rarely contained information about external consultations.

“It [the PHR] is only interesting if something external is happening because you don’t always know about the external things. But as the self-employed colleagues don’t feel bound to this booklet anyway – I am not sure if it makes a lot of sense.” (D5).

Some interviewees speculated that even if external doctors did not document their consultation in the PHR, they might still have read and benefitted from information contained therein. A participant described that external doctors used to call to inquire about patients, saying that after the implementation of the patient-held PHR “this has been very very much reduced, maybe two three phone calls a week as opposed to many per day” (N2).

Surprisingly, one participant was not aware that external doctors were supposed to use the patient-held PHR at all.

### Impact on transfer of information upon leaving the reception center

Some participants saw the transmission of health-related information after the transfer of an asylum seeker to a subsequent accommodation as a key benefit of the PHR because electronic health records are not shared with other institutions.

“We want there to be something that isn’t lost by leaving the current environment because [electronic health records] are lost [...] so that the future doctor no matter where and however he will work, knows that he [the patient] was examined there” (D6).

An opportunity to examine the actual benefit of the PHR in the transfer of patients was offered by the interviews that were conducted at a reception center that had not introduced the PHR. Around one-third of their population had been transferred from reception centers which had implemented PHRs. These participants were very vocal about the benefits of the PHR for the medical care provided at their reception center.

“I am very certain, that it [the PHR] improves the continued care” (N6).

“The health booklet indeed helps us if they [the patients] have been to the doctor in the [previous] reception center and he writes his diagnosis in there or simply the medication. And then we look at that, and we say aha, so he must have been in treatment, or there is a diagnosis. […] So this is […] helpful as an additional communication tool” (D10).

## Discussion

Our results underline the need to improve the availability of health-related information for patients in reception centers and suggest that a patient-held PHR can be a viable solution if three essential requirements are met: First, consistent PHR use by all health personnel involved in the delivery of care to asylum seekers in reception centers must be achieved. In the present case, participants reported using the PHR on a regular basis but stated that adherence was lower among internal and virtually nonexistent among external doctors. Our results suggest that this low adherence might be due to the demanding work environment in reception centers and the existence of parallel systems of medical records. The motivation of health care providers to use the PHR and the associated benefits they perceived were more pronounced in reception centers with a higher degree of internal fragmentation regarding personnel, specialties and medical records. Consistent use of the PHR by external doctors may create added benefit by improving the exchange of medical history across sectoral boundaries. If this is achieved, perceived benefits of the patient-held PHR might increase in reception centers of all levels of fragmentation as they will then be able to access information about external consultations.

Second, patient adherence to the patient-held PHR is essential to the transfer of information. Patient attitudes towards the PHR were generally described as positive, especially in settings where patients were adequately informed about the PHR and where they were asked to fetch their PHR before being treated.

Third, guidelines for implementing a PHR in local settings are needed. Our results show that in the absence of such recommendations, different procedures regarding patient information and integration of the PHR into care delivery routines were followed in each reception center. This led to a wide range of local practices, some of which might negatively affect patient autonomy or even stigmatize asylum seekers with certain medical conditions. Overall, our study shows that the degree of fragmentation in service delivery design and health information systems, consistent use of the PHR by internal and external physicians, sufficient workforce capacity to cope with the additional workload, clear implementation guidelines, electronic health records well integrated with the patient-held PHR and legible handwritings are essential elements for the successful implementation of a patient-held PHR for asylum seekers in reception centers.

### Strengths and limitations

This study is the first evaluation of a patient-held PHR in the asylum seeker population living in reception centers in Germany. It offers valuable insights into the opportunities and shortcomings of the PHR in this setting. Our study is valuable to understanding how the PHR fits into and impacts local care delivery practices and individual behavior of medical staff. These insights are crucial to the design, adaptation, and implementation of a PHR in this complex environment. However, our study only covered the perspectives of care providers and did not include asylum seekers’ view on the PHR. The recruitment of patients for interviews on this issue was ongoing at the time of writing this manuscript. The perspectives of asylum seekers will be triangulated at a later stage with those of healthcare professionals to provide a full picture of perspectives and attitudes among all groups involved in using the PHR. Our study represents an early evaluation of the PHR in this setting. Additional research will be necessary to determine long term sustainability as perspectives and utilization patterns reported by interviewees may change with long term use and embeddedness of the PHR in the facilities’ routines. Moreover, there could be a risk of Hawthorne effect or social desirability bias. This risk may be compounded by the fact that the PHR was developed by the interviewers’ department, although they were not personally involved in the process.

### Patient-held personal health records in other contexts: Experiences, perspectives, and impact

The European Commission and the International Organisation for Migration (IOM) have jointly begun implementing a patient-held PHR for refugees in Greece, Italy, Slovenia, and Croatia between October 2016 and January 2017, but no reports about the experiences from this project have been published yet [21–24]. We are also not aware of recommendations or activities of organisations such as UNHCR, WHO or international NGOs related to patient-held PHRs for refugees (although PHRs are commonly used in many contexts in low- and middle-income countries, including but not limited to refugee settings). Therefore, we can only draw on literature addressing patient-held PHRs in resident populations. Several systematic reviews have examined the attitudes towards and benefits of patient-held PHRs in a broad range of settings [8, 9, 11, 13, 15, 25]. These studies conclude that there is no reliable evidence for the benefits of a PHR regarding the quality and continuity of care. These findings also apply to patients with chronic diseases [13], a subpopulation that study participants identified as profiting most from a well-kept PHR. Our interview results suggest that a PHR can indeed be beneficial for the availability of medical history and the continuity of care in the asylum seeker population. A possible explanation for this divergence of results between migrant and routine care could be that in the reception centers, availability of medical history in the absence of a PHR is often experienced as insufficient and problematic. Therefore, patients and care providers in reception centers stand to benefit more from a PHR than those involved in routine care where baseline satisfaction with availability of medical history is much higher [16]. In our study as well as the wider patient-held PHR literature, a significant barrier to beneficial PHR implementation was found to be low uptake among health professionals. This was reported to be mostly due to time constraints but also worries about confidentiality and litigation as well as the patient-held PHR being regarded as incomplete and therefore unreliable [15, 16, 25]. Existing PHR literature indicates that implementation issues identified in our study are not unique to the asylum seeker context. Indeed, similar implementation challenges and facilitators have been described in the wider implementation research literature.

### Implications for designing the implementation process – Drawing on insights from implementation research

Implementation research seeks to promote “understanding of how to increase integration of evidence-based approaches into routine, real-world practices” [26]. The Consolidated Framework for Implementation Research (CFIR) was developed to describe “what works where and why across multiple contexts” [27] and has since been frequently used to describe factors that influence implementation outcomes [28–30]. The CFIR covers five implementation domains – intervention characteristics, outer and inner setting, characteristics of the individual and the implementation process – with several constructs for each domain [27]. Key implementation challenges and facilitators identified in this study fall squarely under the *inner setting* domain, particularly the concepts of *implementation climate* and *readiness for implementation.*

The acknowledgement of the necessity of a PHR to improve access to health information given the limited communication between providers within and outside of the reception centers is described by the CFIR as *tension for change*. This issue was particularly important in reception centers with a high degree of fragmentation regarding staff, medical specialties or health information systems, indicating that implementation might be facilitated in these settings. In settings of low fragmentation within the institution, where this benefit may not be as pronounced, the need to improve communication with external health professionals may be emphasized instead. On the other hand, *compatibility* with existing workflows, especially regarding health information systems, appeared to be a key hindrance. This factor was compounded by the often perceived lack of support from the facilities (“*relative priority”)*, generally no clear communication of implementation goals *(“goals and feedback”)* as well as time constraints that leave little room to learn to use a new tool *(“learning climate”)*. According to the CFIR, these results are indicative of challenges regarding the reception centers’ *implementation climate*: “the absorptive capacity for change, shared receptivity of involved individuals to an intervention, and the extent to which use of that intervention will be rewarded, supported and expected within their organization” [27]. In addition, interviewees reported little involvement of the authorities responsible for implementation, no provision of additional resources in an already constrained setting as well as very little information about the PHR’s use and implementation process. These three sub-concepts *leadership engagement, available resources* and *access to information* are described as *implementation readiness:* “tangible and immediate indicators of organizational commitment to its decision to implement an intervention”. In this study*, implementation readiness* emerged as the key component affecting implementation outcome, possibly resulting from shortcomings in the implementation process.

Regarding the implementation process itself, the CFIR formulates four concepts – planning, engaging executing, reflecting and evaluating [27]. The formal implementation process described in this paper consisted of local team meetings, written information and informational e-mails. Nevertheless, most participants reported receiving very little information about the PHR, clearly indicating shortcomings particularly in the planning and engaging phase.

To address this issue, we developed a handbook for PHR implementation in reception centers [31]. Local authorities were supplied with this handbook to support the planning of future implementation processes. It includes detailed recommendations and checklists that address the issues mentioned above: timely information and inclusion of health care providers within and outside of the institution through letters, e-mails and informational meetings; formal identification and training of multipliers at the centers; guidelines regarding PHR distribution and example phrases for patient information; identifying and communicating responsibilities; integration into local workflows; a guide for internal staff meetings at the facilities and other implementation aspects [31].

Future implementation processes of patient-held PHRs in the reception center setting should incorporate and expand on these guidelines, taking into account the wide variation between facilities, particularly regarding level of fragmentation, and tailoring the implementation process to each specific setting. As the PHR is designed to be an intersectoral communication tool all sectors involved in the delivery of care for asylum seekers in the region need to be properly informed about the medium and its purpose. A PHR that is not widely used is not useful for patients or the health professionals involved in their care.

### Future prospects: Linking information transfer and quality of care

Future studies should explore the impact of patient-held PHRs on quality of care, both through in-depth qualitative research and long-term quantitative evaluation of clinical outcomes. Our results touch upon issues such as doctor-patient-relationships in the migration context, availability of resources, and working conditions in reception centers as well as problems and solutions regarding language barriers. Each of these aspects should be further explored to better understand the situation in the reception centers and where the introduction of a PHR would fit in – or which obstacles should be addressed before implementing a PHR. In particular, CFIR domains of *characteristics of the individual* and the *outer setting* should be studied in more detail to enable future implementation to address a broader range of implementation factors. Additionally, the implementation process itself should be studied in more detail, with the aim of developing and sharing best practices that could inform PHR introduction in other reception centers and similar institutions across Germany and the European Union. Lastly, the attitudes towards the PHR among the asylum seeker population require more research to provide evidence for recommendations regarding patient information and compliance.

## Conclusion

A patient-held PHR can be a means to improve the availability of health-related information in reception centers provided that a context-sensitive implementation process is realized to overcome the identified barriers. The present case shows how an incomprehensive implementation process can result in low adherence to the patient-held PHR and give rise to substantive skepticism about its usefulness. The benefit of a PHR could be most pronounced in reception centers that are particularly fragmented in terms of systems of electronic health records and medical personnel and for patients whose treatment requires the long-term involvement of multiple care providers. Our findings can inform the design and implementation of PHRs for refugees and asylum seekers in other regions of Germany and Europe. They indicate that a patient-held PHR could be a simple tool to improve continuity and quality of care in the asylum seeker population and may serve as the basis for further exploration of how this potential can be harnessed best.

## Additional files

Additional file 1: Interview guide. (ZIP 30 kb)

Additional file 2: Code System. (DOCX 22 kb)

## Abbreviations

CFIR: Consolidated Framework for Implementation Research
IOM: International Organization for Migration
NGO: Non-Governmental Organization
PHR: Personal Health Record
PHV: Patrick-Henry Village
UNHCR: United Nations High Commissioner for Refugees
WHO: World Health Organization

## References

[1] European Asylum Support Office. Latest asylum trends - June 2017. https://www.easo.europa.eu/latest-asylum-trends. Accessed 11 July 2018.

[2] Bradby H, Humphris R, Newall D, Phillimore J (2015). Public health aspects of migrant health: a review of the evidence on health status for refugees and asylum seekers in the European region.

[3] Priebe S, Sandhu S, Dias S, Gaddini A, Greacen T, Ioannidis E, et al. Good practice in health care for migrants: views and experiences of care professionals in 16 European countries. BMC Public Health 2011;11(1):187. 10.1186/1471-2458-11-187 PMC3071322 21439059

[4] Bozorgmehr K, Wahedi K, Noest S, Szecsenyi J, Razum O. Infectious disease screening in asylum seekers: range, coverage and economic evaluation in Germany, 2015. Eur Secur. 2017;22(40) 10.2807/1560-7917.ES.2017.22.40.16-00677 PMC5710125 29019315

[5] Bozorgmehr K, Razum O, Gewalt S, Nöst S. Germany: optimizing service provision to asylum-seekers. In: WHO compendium of good practice on health system responses to migration in Europe. Copenhagen. in press

[6] Bozorgmehr K, Nöst S, Thaiss HM, Razum O (2016). Die gesundheitliche Versorgungssituation von Asylsuchenden : Bundesweite Bestandsaufnahme über die Gesundheitsämter. Bundesgesundheitsblatt - Gesundheitsforsch - Gesundheitsschutz.

[7] Warner JP, King M, Blizard R, McClenahan Z, Tang S (2000). Patient-held shared care records for individuals with mental illness: randomised controlled evaluation. Br J Psychiatry.

[8] Schoevers MA, van den Muijsenbergh ME, Lagro-Janssen AL. Patient-held records for undocumented immigrants: a blind spot. A systematic review of patient-held records. Ethn Health. 2009;14(5):497-508. 10.1080/13557850902923273 19462264

[9] Archer N, Fevrier-Thomas U, Lokker C, McKibbon KA, Straus SE (2011). Personal health records: a scoping review. J Am Med Informatics Assoc.

[10] Jerden L, Hillervik C, Hansson A-C, Flacking R, Weinehall L (2006). Experiences of Swedish community health nurses working with health promotion and a patient-held health record. Scand J Caring Sci.

[11] Nguyen M, Lennox N, Ware R (2014). Hand-held health records for individuals with intellectual disability: a systematic review. J Intellect Disabil Res.

[12] Lecouturier J, Crack L, Mannix K, Hall RH, Bond S (2002). Evaluation of a patient-held record for patients with cancer. Eur J Cancer Care (Engl).

[13] Ko H, Turner T, Jones C, Hill C. Patient-held medical records for patients with chronic disease: a systematic review. BMJ Qual Saf 2010;19(5):e41–e41. 10.1136/qshc.2009.037531 20511601

[14] Williams JG, Cheung W-Y, Chetwynd N, Cohen DR, El-Sharkawi S, Finlay I (2001). Pragmatic randomised trial to evaluate the use of patient held records for the continuing care of patients with cancer. Qual Heal Care.

[15] Gysels M, Richardson A, Higginson IJ (2007). Does the patient-held record improve continuity and related outcomes in cancer care: a systematic review. Health Expect.

[16] Cornbleet MA, Campbell P, Murray S, Stevenson M, Bond S (2002). Patient-held records in cancer and palliative care: a randomized, prospective trial. Palliat Med.

[17] Chen HT (2016). Interfacing theories of program with theories of evaluation for advancing evaluation practice: reductionism, systems thinking, and pragmatic synthesis. Eval Program Plann.

[18] Setzer Verlag. tip doc.http://www.setzer-verlag.com/epages/79584208.sf/de_DE/?ObjectPath=/Shops/79584208/Categories/Category1. Accessed 23 July 2017.

[19] Aktionsbündnis “Sichere Arzneimittelanwendung Rhein-Neckar-Kreis Heidelberg.” Mein Plan - Aktionsbündnis “Sichere Arzneimittelanwendung Rhein-Neckar-Kreis Heidelberg”. http://www.nimmsrichtig.de/. Accessed 23 July 2017.

[20] Holliday A. Doing and writing qualitative research. 2nd ed. SAGE Publications Ltd; 2007. 60–112 p.

[21] International Organization for Migration, European Commission. Health Assessment of Refugees and Migrants in the EU/EEA - Handbook for professionals. http://ec.europa.eu/dgs/health_food-safety/docs/personal_health_handbook_english.pdf. Accessed 21 July 2017.

[22] International Organization for Migration, European Commission. Personal Health Record. http://ec.europa.eu/dgs/health_food-safety/docs/personal_health_record_english.pdf. Accessed 21 July 2017.

[23] Consumers, Health, Agriculture and Food Executive Agency EC. Re-Health final conference took place on 15 May 2017 in Brussels. http://ec.europa.eu/chafea/news/news503.html. Accessed 21 July 2017.

[24] International Organization for Migration. Re-Health: Implementation of the PHR. Available from: http://re-health.eea.iom.int/implementation-phr-deliverables. Accessed 23 July 2017.

[25] Hawley G, Janamian T, Jackson C, Wilkinson SA (2014). In a maternity shared-care environment, what do we know about the paper hand-held and electronic health record: a systematic literature review. BMC Pregnancy Childbirt.

[26] Colditz GA, Emmons KM. The promise and challenges of dissemination and implementation research. In: Dissemination and Implementation Research in Health: Translating Science into Practice 2nd ed. 2018:1–19.

[27] Damschroder LJ, Aron DC, Keith RE, Kirsh SR, Alexander JA, Lowery JC (2009). Fostering implementation of health services research findings into practice: a consolidated framework for advancing implementation science. Implement Sci.

[28] Kirk MA, Kelley C, Yankey N, Birken SA, Abadie B, Damschroder L. A systematic review of the use of the consolidated framework for implementation research. Implement Sci. 2016;11(1) 10.1186/s13012-016-0437-z PMC4869309 27189233

[29] Birken SA, Powell BJ, Shea CM, Haines ER, Kirk MA. Leeman J, et al. Criteria for selecting implementation science theories and frameworks : results from an international survey. 2017:1–9. 10.1186/s13012-017-0656-y PMC5663064 29084566

[30] Nilsen P (2015). Making sense of implementation theories, models and frameworks. Implement Sci.

[31] Bozorgmehr K, Ziegler S, Noest S, Jahn R, Strassner C. Das persönliche Gesundheitsheft für Asylsuchende - Handbuch zur Einführung und Nutzung (Version 1.0). Department of General Practice and Health Services Research, University Hotpital Heidelberg, 2017. https://www.gesundheitsheft.info/downloads/Handbuch_Einfuehrung_Gesundheitsheft.pdf?ts=1529507342667. Accessed 20 June 2018.

