# Supplementary material for: Early evaluation of experiences of health care providers in reception centers with a patient-held personal health record for asylum seekers: a multi-sited qualitative study in a German federal state
Source: Global Health. 2018 Jul 20;14:71. doi: 10.1186/s12992-018-0394-1 (PMC6054720; doi:10.1186/s12992-018-0394-1)
Supplement: Supplementary file 1 — Interview guide. (ZIP 30 kb) [file 12992_2018_394_MOESM1_ESM.zip › InterviewGuide_FINAL_DE_EN_nurses.docx]

**Evaluation Gesundheitsheft – Übersicht Interviewleitfaden**

Kurze Einführung zum Heft, Danke für die Teilnahme, Einverständnis zur Aufnahme des Interviews?

1. Wie sind denn Ihre **persönlichen Erfahrungen** mit dem Gesundheitsheft?
2. Sie setzen das Heft ja jetzt schon einige Zeit ein, was hat sich denn an den **Abläufen hier in der Einrichtung geändert**?
   1. Wie war denn die **Einführung des Heftes?**
   2. Was hat **gut funktioniert?** Was hat **nicht so gut funktioniert?**
   3. Wie sind Sie denn mit den Schwierigkeiten **umgegangen**? Gab es **Lösungen?**
   4. Was würden Sie denn anderen Einrichtungen empfehlen, die das Heft nun erst neu einführen?
3. Wie sieht es denn **an der Anmeldung** hier, mit dem Gesundheitsheft aus?
   1. Wenn ein Patient ein **neues Heft** von Ihnen bekommt, was sagen sie ihm/ihr dazu?
   2. Heft **vergessen?** Nachfrage nach Heft?
   3. **Wie erleben Sie die Patienten im Umgang mit dem Gesundheitsheft? (oder unten)**
   4. Was funktioniert **gut**, was **nicht so**?
4. (Wie erleben Sie die **Patienten im Umgang mit dem Gesundheitsheft**?)
5. Welchen Eindruck haben Sie davon, wie **die Ärzte** mit dem Heft umgehen? Das Heft benutzen?
6. Welchen Eindruck haben Sie, wie die **Ärzte außerhalb der Ambulanz** mit dem Heft umgehen?
   1. Ein Ziel des Heftes war es ja, das Information besser weitergegeben werden zwischen den Versorgern, wie würden Sie das einschätzen?
7. Können Sie sich **negative oder unbeabsichtigte Folgen** des Heftes vorstellen?
8. Wo sehen Sie **Vorteile** des Gesundheitsheftes?
9. Wir interessieren uns auch für Ihre **Rückmeldungen und Ideen zu dem Heft selbst**. Ich habe hier noch einmal ein Exemplar mitgebracht und würde das gerne mit Ihnen durchblättern. (incl. Nachfragen nach Nutzung best. Seiten z.B. zur Termineintragung Folgetermine)

Ausstieg: Jetzt haben wir schon viel zum Gesundheitsheft gesagt, gibt es noch etwas, das sie noch ansprechen wollen, was bisher noch nicht Thema war?

**Evaluation PHR – interview guide (English translation) - nurses**

Brief introduction to the PHR, thanks for participation, agreement to record the interview

1. What are your **personal experiences** with the PHR?
2. You have already used the PHR for some time now, what **changes to the processes have you seen in this institution**?
   1. How did the **introduction of the PHR** go?
   2. What **worked well**? What **didn’t work so well**?
   3. How did you **approach difficulties**? Were there any **solutions**?
   4. What advice would you give other institutions introducing the PHR?
3. How does the **registration** with the PHR work?
   1. When a patient receives a **new PHR**  from you, what do you tell them?
   2. What happens when someone has **forgotten** their PHR? Do you ask for it?
   3. **How do patients experience the use of the PHR?** (Here or later)
   4. What works **well**, what **doesn’t work well**?
4. (**How do patients experience the use of the PHR?)**
5. What are your impressions on how **physicians** use the PHR?
6. What are your impressions on how **physicians outside of the facility** use the PHR?
   1. One of the goals of the PHR was to improve information transfer between providers, would you say this worked?
7. Can you think of any **negative or unintended consequences** of the PHR?
8. Where do you see the **benefits** of the PHR?
9. We are also interested in your **feedback and ideas for the PHR itself**. I have brought an example with me and would like to look through it with you. (including questions about the use of specific pages, e.g. for entry of appointments)

Exit: We have now talked a lot about the PHR, is there anything else you would like to add that we haven’t yet talked about?
