# Supplementary material for: Early evaluation of experiences of health care providers in reception centers with a patient-held personal health record for asylum seekers: a multi-sited qualitative study in a German federal state
Source: Global Health. 2018 Jul 20;14:71. doi: 10.1186/s12992-018-0394-1 (PMC6054720; doi:10.1186/s12992-018-0394-1)
Supplement: Supplementary file 1 — Interview guide. (ZIP 30 kb) [file 12992_2018_394_MOESM1_ESM.zip › InterviewGuide_FINAL_DE_EN_physicians.docx]

**Evaluation Gesundheitsheft – Übersicht Interviewleitfaden**

Kurze Einführung zum Heft, Danke für die Teilnahme, Einverständnis zur Aufnahme des Interviews?

1. Als erstes würde mich interessieren, welche Rolle **spielen denn Informationen** überhaupt in der Behandlung, ganz allgemein?
   1. Welche Bedeutung haben denn **schriftliche Informationen?**
   2. Was ist, wenn **keine schriftlichen Informationen vorliegen?**
   3. Wie gehen Sie denn mit den **Aussagen der Patienten** um?
2. Wie sind denn Ihre **persönlichen Erfahrungen** mit dem Gesundheitsheft?
3. Wie sieht das denn in der **konkreten Behandlungssituation mit Gesundheitsheft hier bei Ihnen** aus?
   1. Wenn ein Patient ein **neues Heft** von Ihnen bekommt / mit einem **leeren Heft** zu Ihnen kommt? (Wie erklären Sie das?)
   2. Wie ist das bei einem Patienten, der schon **länger ein Heft hat**?
   3. Möglich: Heft vergessen? Nachfrage nach Heft?
4. Welchen Eindruck haben Sie davon, wie Ihre Kollegen mit dem Heft umgehen?
5. Sie setzen das Heft ja jetzt schon einige Zeit ein, was hat sich denn an den **Abläufen hier in der Einrichtung geändert**?
   1. Wie war denn die **Einführung des Heftes?**
   2. Was hat **gut funktioniert?** Was hat **nicht so gut funktioniert?**
   3. Wie sind Sie denn mit den Schwierigkeiten **umgegangen**? Gab es **Lösungen?**
   4. Was würden Sie denn anderen Einrichtungen empfehlen, die das Heft nun erst neu einführen?
6. Was denken Sie, was hat sich denn seit der Einführung des Gesundheitsheftes **für die Patienten verändert**? (s. evtl. weiter mit Frage 8. oder Überleitung zu 7.)
7. Was denken Sie, was hat das Heft **in der Weiterversorgung der Patienten verändert**?
   1. Ein Ziel des Heftes war es ja, das **Information besser weitergegeben werden zwischen den Versorgern,** wie würden Sie das einschätzen?
   2. Können Sie sich auch **negative oder unbeabsichtigte Folgen** des Heftes vorstellen?
   3. (Bei Lamentieren: Wo sehen Sie **Vorteile** des Gesundheitsheftes?)
8. Wie erleben Sie denn die **Patienten im Umgang** mit dem Gesundheitsheft?
9. Wir interessieren uns auch für Ihre **Rückmeldungen und Ideen zu dem Heft selbst**. Ich habe hier noch einmal ein Exemplar mitgebracht und würde das gerne mit Ihnen durchblättern. (incl. Nachfragen nach Nutzung best. Seiten z.B. zur Termineintragung Folgetermine)

Ausstieg: Jetzt haben wir schon viel zum Gesundheitsheft gesagt, gibt es noch etwas, das sie noch ansprechen wollen, was bisher noch nicht Thema war?

**Evaluation personal health record – interview guide (English translation) - physicians**

Brief introduction to the PHR, thanks for participation, agreement to record the interview

1. First I would be interested to hear **what role information plays** in the treatment of patients overall
   1. What importance does **written information** have?
   2. What happens, when you **don’t have any written information**?
   3. How do you handle what the **patients tell you** about their illness?
2. What are your **personal experiences** with the PHR?
3. So how is the health information booklet used **during a specific consultation** with you?
   1. What do you do when a patient receives a **new PHR** from you or comes to you with an **empty PHR**?
   2. What are your experiences with patients that have **already had their PHR for some time**?
   3. What happens when someone has **forgotten** their PHR? Do you ask for it?
4. What are your impressions of how your colleagues use the PHR?
5. You have already used the PHR for some time now, what **changes to the processes have you seen in this institution**?
   1. How did the **introduction of the PHR** go?
   2. What **worked well**? What **didn’t work so well**?
   3. How did you **approach difficulties**? Were there any **solutions**?
   4. What advice would you give other institutions introducing the PHR?
6. What do you think has changed for the patients since the introduction of the PHR?
7. What has changed for the **continued care for patients** since the introduction of the PHR?
   1. One of the goals of the PHR was to improve information transfer between providers, would you say this worked?
   2. Can you think of any **negative or unintended consequences** of the PHR?
   3. If there are many complaints: Where do you see the benefits of the PHR?
8. **How do patients experience the use of the PHR?**
9. We are also interested in your **feedback and ideas for the PHR itself**. I have brought an example with me and would like to look through it with you. (including questions about the use of specific pages, e.g. for entry of appointments)

Exit: We have now talked a lot about the PHR, is there anything else you would like to add that we haven’t yet talked about?
