# Supplementary material for: Early evaluation of experiences of health care providers in reception centers with a patient-held personal health record for asylum seekers: a multi-sited qualitative study in a German federal state
Source: Global Health. 2018 Jul 20;14:71. doi: 10.1186/s12992-018-0394-1 (PMC6054720; doi:10.1186/s12992-018-0394-1)
Supplement: Supplementary file 2 — Code System. (DOCX 22 kb) [file 12992_2018_394_MOESM2_ESM.docx]

| **Codes** | | | **No of codings** |
| --- | --- | --- | --- |
| Wichtigkeit/Relevanz Informationen | | | 32 |
| Einführung des Heftes | | | 0 |
|  | Einführungshindernisse | | 43 |
|  | Beschreibung | | 13 |
|  | Lösungsstrategien/Tipps | | 20 |
| Kontextvariablen | | | 0 |
|  | institutionell/organisatorisch | | 83 |
|  | individuell | | 51 |
|  | Sonstiges | | 6 |
| Ablauf bez. Heft | | | 17 |
| Patientenführung | | | 38 |
| Patientenumgang Heft | | | 44 |
| Nutzung | | | 0 |
|  | Eigene Nutzung | | 0 |
|  |  | Informationen selbst eintragen | 38 |
|  |  | Eigene Dokumentation ausdrucken | 7 |
|  |  | Unterlagen sammeln | 14 |
|  |  | Informationen entnehmen & nutzen | 30 |
|  |  | Anderes | 9 |
|  | Kollegen | | 0 |
|  |  | Kollegen extern | 30 |
|  |  | Kollegen intern | 28 |
|  | Allgemein | | 9 |
| Nutzenbewertung | | | 0 |
|  | Allgemeine Aussagen | | 26 |
|  | Verbesserte medizinische Versorgung | | 5 |
|  | Verfügbarkeit Informationen generell | | 14 |
|  | Sprachbarriere umgehen | | 7 |
|  | Interne Kommunikation & Abläufe | | 0 |
|  |  | Positiv | 19 |
|  |  | Negativ/Neutral | 10 |
|  | Externe Kommunikation | | 0 |
|  |  | Positiv | 13 |
|  |  | Negativ/Neutral | 5 |
|  | Transfer | | 1 |
|  |  | Positiv | 24 |
|  |  | Negativ | 3 |
|  | Anderer Nutzen | | 0 |
|  |  | Negativ | 22 |
|  |  | Positiv | 5 |
| Layout/Design | | | 5 |
| Sonstige Themen | | | 1 |
| All | | | 687 |

**Code System – Original German Version**

**Code System – English**

| **Codes** | | | | **No of codings** |
| --- | --- | --- | --- | --- |
| Importance / Relevance of Information | | | | 32 |
| PHR Implementation | | | | 0 |
|  | Implementation barriers | | | 43 |
|  | Implementation process | | | 13 |
|  | Solutions / Advice | | | 20 |
| Context | | | | 0 |
|  | Institutional | | | 83 |
|  | Individual | | | 51 |
|  | Other | | | 6 |
| PHR routines | | | | 17 |
| Patient management | | | | 38 |
| Patient behavior regarding PHR | | | | 44 |
| PHR Utilisation | | | | 0 |
|  | Participants | | | 0 |
|  |  | | Reporting in the PHR | 38 |
|  |  | | Printing ePHR | 7 |
|  |  | | Collecting documents | 14 |
|  |  | | Accessing/using information | 30 |
|  |  | | Other | 9 |
|  | Colleagues | | | 0 |
|  |  | | External colleagues | 30 |
|  |  | | Internal colleagues | 28 |
|  | Other | | | 9 |
| Benefits | | | | 0 |
|  | General remarks | | | 26 |
|  | Improved medical care | | | 5 |
|  | Availability medical history | | | 14 |
|  | Circumventing language barriers | | | 7 |
|  | | Internal communication & routines | | 0 |
|  |  | | Positive | 19 |
|  |  | | Negative/Neutral | 10 |
|  | External communication | | | 0 |
|  |  | | Positive | 13 |
|  |  | | Negative/Neutral | 5 |
|  | Transfer | | | 1 |
|  |  | | Positive | 24 |
|  |  | | Negative | 3 |
|  | Other effects | | | 0 |
|  |  | | Negative | 22 |
|  |  | | Positive | 5 |
| Layout/Design | | | | 5 |
| Other topics | | | | 1 |
| All | | | | 687 |
